# Supplementary material for: Short-term activation of the Jun N-terminal kinase pathway in apoptosis-deficient cells of Drosophila induces tumorigenesis
Source: Nat Commun. 2018 Apr 18;9:1541. doi: 10.1038/s41467-018-04000-6 (PMC5906466; doi:10.1038/s41467-018-04000-6)
Supplement: Supplementary file 1 — Supplementary Information [file 41467_2018_4000_MOESM1_ESM.pdf]

Short-term activation of the Jun-N terminal Kinase pathway in apoptosis-deficient cells of *Drosophila* induces tumorigenesis

Pinal et al

Supplementary Fig. 1

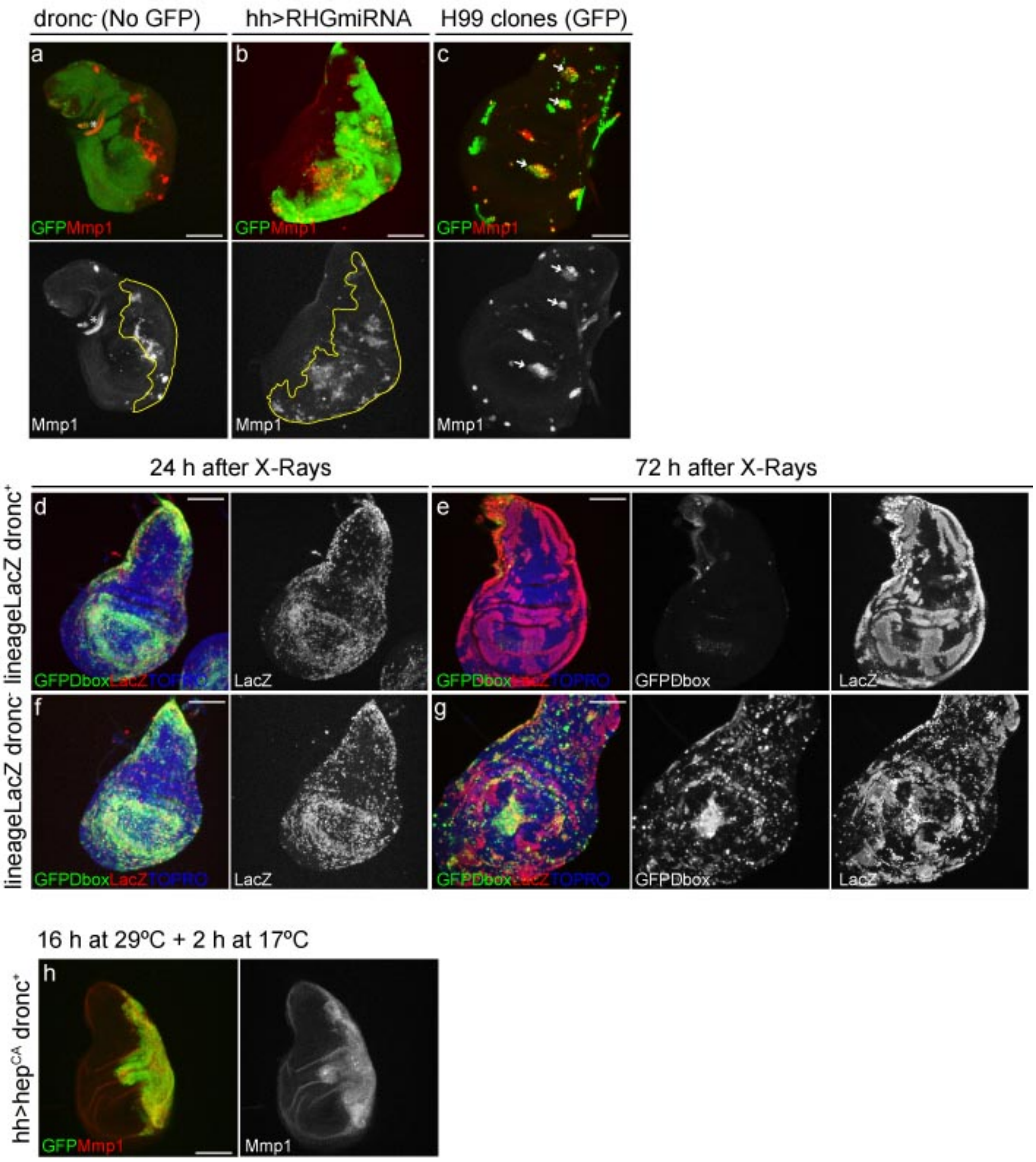

Maintenance of JNK activity in apoptosis deficient cells and lineage tracing of those cells **a-c** Persistence of JNK activity 96 h after irradiation in discs of three apoptosis-deficient genotypes. **a** Wing disc of genotype *en-Gal4/UAS-Flp;dronc*^{i29}*FRT2A/M(3)67C FRT2AUbi-GFP* (n=10). *M*$^{+}$ *dronc*^{i29} clones cover most of the posterior compartment and lose the GFP mark. The anterior compartment remains *dronc*$^{+}$ and retains the GFP label. In this experiment we used Mmp1 (red) as marker of JNK activity. Note that the red label is restricted to the posterior compartment. The red mark in the anterior compartment (asterisk) corresponds to trachea, which are also labelled by Mmp1. **b** Wing disc of genotype *UAS-RHG-miRNA/+;hh-Gal4UAS-GFP/+* (n=6). The *UAS-RHGmiRNA* construct expresses a microRNA that suppresses the proapoptotic genes *reaper*, *hid* and *grim*. Activation of JNK is detected only in the posterior compartment (green) with Mmp1 staining (red). **c** Wing disc of *genotype hs-Flp tub-Gal4UAS-GFP Df(3L)H99FRT2A/FRT2Atub-Gal80* (n=7) containing clones homozygous for *DfH99* (green), a deletion removing *reaper*, *hid* and *grim*. JNK activity

as revealed by Mmp1 (red) is detected in the clones (arrows). **d-g** Wing discs of genotype: *UAS-GFPDbox/act<stop>LacZ;puc-Gal4/UAS-flpdronc*^{i24} (**d**) 24 h (n=7) and (**e**) 72 h (n=19) after irradiation and *UAS-GFPDbox/act<stop>LacZ;puc-Gal4 dronc*^{i29}*/UAS-flpdronc*^{i24} (**f**) 24 h (n=6) and (**g**) 72 h (n=21) after irradiation. After X-Rays JNK activation induces the expression of *puc-Gal4*, which activates the *UAS-GFPDbox* and the *UAS-flp*, therefore inducing the recombination in the lineage cassette that labels cells indelibly. 24 h after irradiation most cells positive for GFPDbox are also labelled with *β-Gal*, both in *dronc*$^{+}$ (**d**) and *dronc*$^{-}$ (**f**) discs. Nevertheless, 72 h after irradiation in the *dronc*$^{+}$ control (**e**) many *β-Gal* (red) positive cells are still present in the tissue, but only cells in the proximal region maintain *GFPDbox* (green). In contrast, in the *dronc*$^{-}$ mutant (**g**) most cells with β-Gal (red) are also positive for GFPDbox (green). **h** Control disc for the pulse experiment, genotype; *UAS-hep*^{CA}*/UAS-GFPtubGal80*^{ts}*;hhGal4 dronc*^{i29}/+, showing activation of JNK (Mmp1, red) in the posterior compartment (green) 2 h after a pulse of 16h at 29ºC. All scale bars are 100 μm.

Supplementary Fig. 2

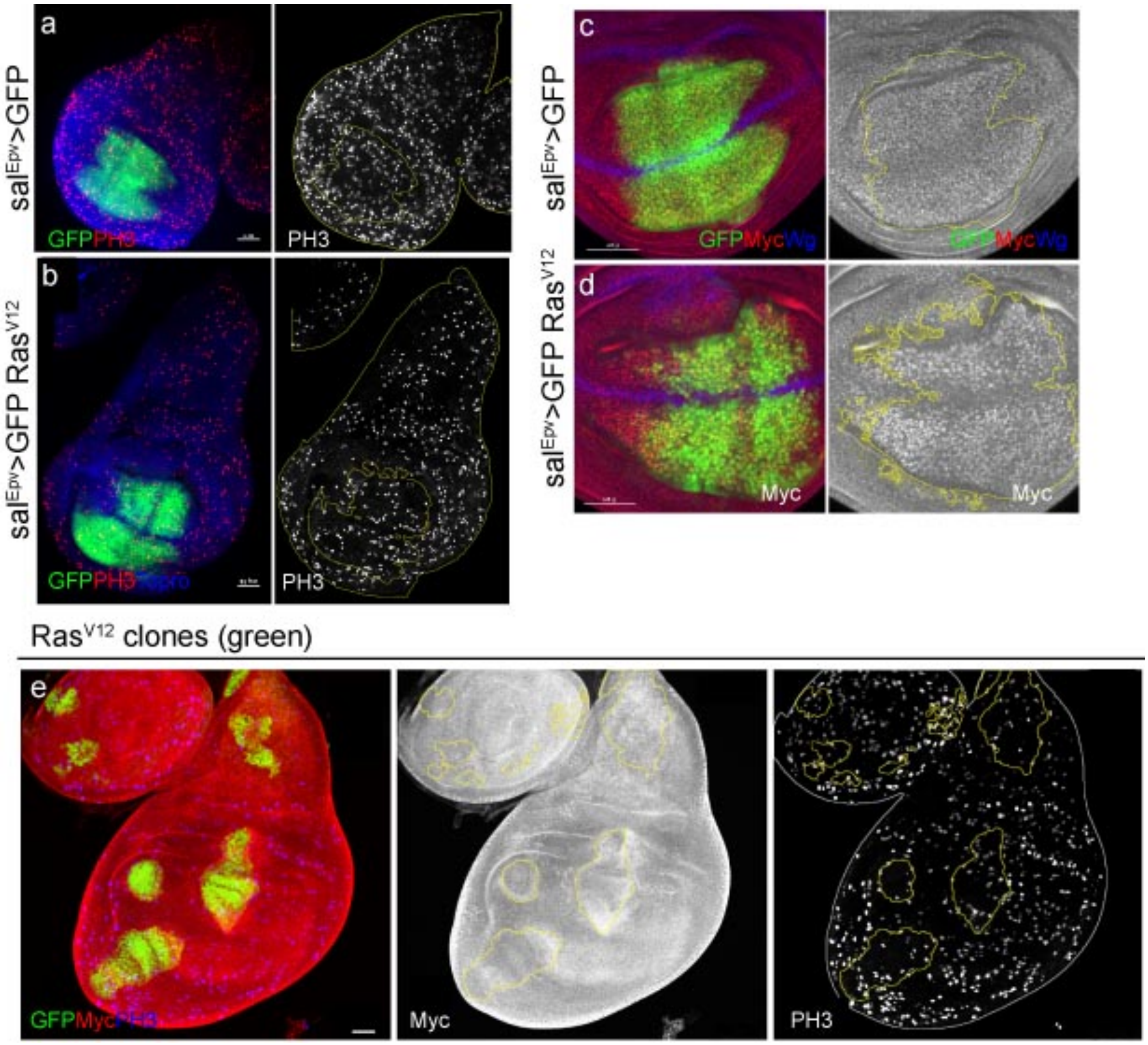

Growth of *Ras*^{V12} tissue. **a**-**d** Images of non-irradiated discs of control genotype *sal-Gal4UAS-GFP* (**a**, **c**) and experimental genotype *sal*^{EPV}*-Gal4 UAS-GFP/UAS-Ras*^{V12}(**b**, **d**). Note in **a** (n=8) and **b** (n=23) that the size of the Sal domain (green) is not significantly affected by the over-expression of Ras. Also, PH3 (red) staining indicates a similar proliferation rate. **c** (n=13) and **d** (n=9) panels show that Myc (red) levels are higher in *Ras*^{V12} than in normal cells, even though the Sal domain is not increased in size. **e** Clones of *Ras*^{V12} expressing cells induced in discs (n=12) of genotype *hsflptub-Gal4 UAS-GFP;FRT40A tub-Gal80/FRT40A;UAS-Ras*^{V12}/+. The *Ras*^{V12} clones (green) show high levels of Myc (red) in some cells but, as indicated by the PH3 staining (blue), they grow at the same rate as the surrounding tissue. All scale bars are 57 µm.

Supplementary Fig. 3

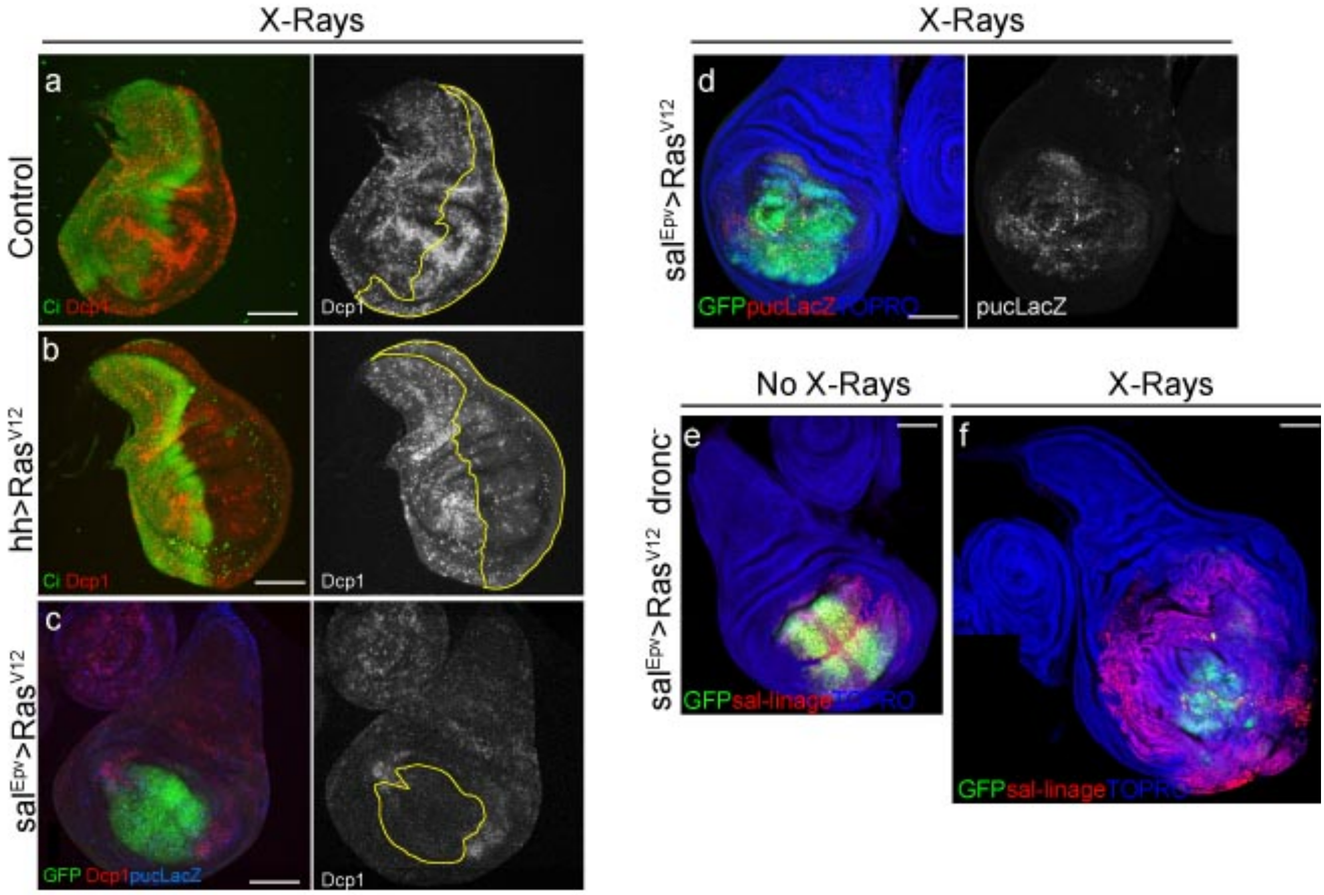

Response of Ras^{V12} tissue to irradiation **a**-**c** Images of wing discs of genotypes: **a** *tub-Gal80^{ts}/+;hh-Gal4/+* (n=10), **b** t*ub-Gal80^{ts}/UAS-Ras^{V12};hh-Gal4/+* (n=10) and **c** *sal^{Epv}-Gal4UAS-GFP/UAS-Ras^{V12};pucLacZ/+* (n=11) 24 h after irradiation. In **a** and **b**, the posterior compartment is marked by the lack of Ci expression (green), whereas in **c** the Ras^{V12} tissue is labelled green with GFP. Note that in **b** and **c** the Ras^{V12} tissue shows a strong reduction in the apoptosis response to X-rays, as monitored by staining with the Dcp1 caspase (red). **d** Image of a wing disc of the genotype: *sal^{Epv}-Gal4UAS-GFP/UAS-Ras^{V12};pucLacZ/+* that shows persistent JNK activity (red) in the Sal domain (green) 72 h after irradiation. In non-Ras^{V12} cells JNK activity is depleted shortly after irradiation (see figure 1). **e**, **f**. Wing discs of the genotype *sal-Gal4UAS-GFP/act<stop>lacZUAS-Flp;dronc^{i29}UAS-Ras^{V12}/dronci^{24}* non-irradiated **e** (n=6) and 96 h after irradiation **f** (n=9). In this experiment we have used the *act>stop>lacZ* cassette to trace the lineage (red) of the cells in the Sal domain. The only difference between the two discs is that the one in F has been irradiated. Yet, there is a large difference in size, especially in the wing pouch, illustrated by the *lacZ*-expressing cells in red. Note that the notum area, in the top left part of the images is similar in both discs. All scale bars are 100 μm.
